# Active Fraction of *Tillandsia usneoides* Induces Structural Neuroplasticity in Cortical Neuron Cultures from Wistar Rats

**DOI:** 10.3390/ijms262311668

**Published:** 2025-12-02

**Authors:** Wilson Leonardo Villarreal Romero, Jhon J. Sutachan, Geison Modesti Costa, Sonia Luz Albarracín

**Affiliations:** 1 Phytochemical Research Group, Faculty of Sciences, Pontificia Universidad Javeriana, Bogotá 110231, Colombia; modesticosta.g@javeriana.edu.co; 2 Experimental and Computational Biochemistry Group, Neurobiochemistry Laboratory, Faculty of Sciences, Pontificia Universidad Javeriana, Bogotá 110231, Colombia; jsutachan@javeriana.edu.co

**Keywords:** neuroplasticity, dendritogenesis, flavonoids, *Tillandsia usneoides*

## Abstract

Neuroplasticity refers to the nervous system’s ability to modify its structure and function in response to intrinsic and extrinsic stimuli. Impairments in this capacity are associated with various neurological disorders, underscoring the need for therapies that preserve or enhance neuronal plasticity. Medicinal plants offer a promising source of bioactive compounds with neuroplastic properties and neuroprotective potential. In this work, we report the chemical and neuroplastic properties of *Tillandsia usneoides*, a medicinal native plant from America. Ethanolic extracts (EtOH) of leaves and stems, along with subfractionated ethyl acetate (EtOAc) and hydroethanolic (H_2_O:EtOH) extracts, were analyzed using High-Performance Thin-Layer Chromatography (HPTLC) and Ultra-Performance Liquid Chromatography coupled with a Diode Array Detector (UPLC-DAD), revealing the presence of 14 phenolic acids, 6 flavonoids, and triterpene. Additionally, functional analysis using Sholl analysis showed that the EtOAc fraction of *Tillandsia usneoides* significantly enhanced structural plasticity in vitro, increasing both dendritic branching and dendrite length at concentrations between 0.03 and 1 μg mL^−1^, likely through the activation PI3K/Akt and ERK1/2 signaling pathways. Together, our results suggest that *Tillandsia usneoides* contains bioactive polar metabolites capable of inducing neuronal structural plasticity.

## 1. Introduction

Neuroplasticity is a process that involves adaptive structural and functional changes in the brain, leading to the formation of new connections between neurons in response to internal and external factors [1,2,3]. These changes involve the remodeling of dendrites, the formation and elimination of spines, and the strengthening or weakening of synapses, mechanisms related to cognition, including learning and memory [1,4,5]. It has been reported that neuroplasticity can be induced by activity. As a result, structural and functional neuroplastic properties can be remodeled throughout life, largely depending on experience and stimuli (activity-dependent) [1,6].

This process is regulated by the activation of several signaling pathways, including the extracellular signal-regulated kinase (Erk) pathway, the mitogen-activated protein kinase (MAPK) pathway, phosphoinositide 3-kinase/protein kinase B (PI3K-Akt) pathway, and the calcium/calmodulin-dependent protein kinase II (CaMKII) pathway [7,8,9]. These pathways regulate neurogenesis, dendritogenesis, spinogenesis, and synaptogenesis [2,3,10], as well as neuronal growth and survival [8,10,11,12]. Regarding the regulation of these neuroplastic processes, it has been reported that plant extracts and secondary metabolites from medicinal species can modulate these pathways and, consequently, induce neuroplasticity [13]. Among the most relevant compounds are phenolic derivatives, primarily flavonoids, some of which have demonstrated neuroprotective and neurotrophic effects. These molecules may prevent the loss of neuroplasticity in the early stages of neurodegenerative diseases and reduce the neurotoxic effects associated with cancer treatments, particularly those induced by chemotherapy [14,15]. Some flavonoids can induce the formation of dendrites and dendritic spines, which are critical structures for synaptic transmission [8,13,16,17,18,19]. In addition, these compounds may act as neurotrophin analogs, supporting neuronal survival and growth, thereby contributing to both recovery and the prevention of neurodegeneration [2,13,19,20,21,22]. However, medicinal plants with secondary metabolites capable of inducing structural and functional neuroplasticity have been poorly studied, and even less is known about the mechanisms. Advancing this knowledge could lead to the identification of candidate mixtures of molecules that can regulate structural neuroplasticity [23,24].

*Tillandsia usneoides* species, a monocotyledonous plant belonging to the *Bromeliaceae* (Linnaeus) family endemic to America, is characterized by being epiphytic and having leaves covered with trichomes, allowing it to obtain water, minerals, and other atmospheric nutrients [25,26]. The English name for *Tillandsia usneoides* is Spanish moss, Grandfather’s Whiskers, Old Man’s Beard, or air plant. Their species are distributed throughout the Neotropics, from Chile to the southeastern United States. It is widely distributed in Colombia and can grow at different temperature ranges up to 3000 meters above sea level, but generally below 1800 meters above sea level [27,28]. Traditional medicine has been reported to utilize the entire plant to treat various conditions, including heart conditions, rheumatism, bronchitis, fever, ulcers, diabetes, and to possess diuretic properties. At the level of the nervous system, it is also used as an analgesic and for the treatment of epilepsy [26,29]. In addition, compounds such as cycloartane-type triterpenes and methoxylated flavonones have been reported [26,30,31,32,33,34]. This work aimed to determine the chemical composition of *Tillandsia usneoides* and evaluate its biological activity in inducing neuroplasticity in cortical neurons of rat embryos.

## 2. Results

### 2.1. Qualitative Analysis of Tillandsia Usneoides by High Performance Thin Layer Chromatography (HPTLC)

**Figure 1:**
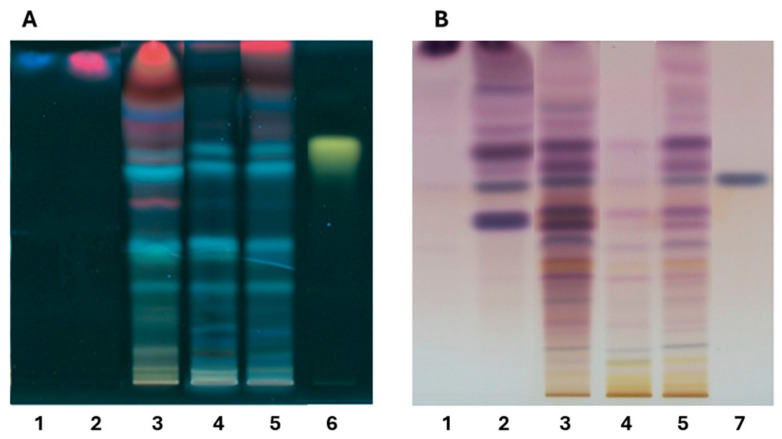
Chromatographic profile HPTLC. (**A**) Detection with natural reagent/UV 365 nm. (**B**) Detection with Sulfuric Vanillin/100 °C. Fractions Hex (1), DCM (2), EtOAc (3), H_2_O:EtOH (4), EtOH (5), Quercetin (6) and Sitosterol (7).

HPTLC analysis of the *Tillandsia usneoides* extract and fractions, developed with the natural reagent and observed under UV light at 365 nm, revealed yellow-orange and blue bands, characteristic of flavonoids with a flavonol core and phenolic acids, respectively. These compounds were predominantly present in the ethanol (EtOH) extract and in fractions of medium and high polarity, including the EtOAc and H_2_O:EtOH fractions (Figure 1A). In contrast, development with vanillin followed by heating revealed terpenoid-type compounds (blue bands) and sterol-type compounds (purple bands) [35], primarily in the DCM and EtOAc fractions (Figure 1B). These fractions showed particularly intense bands, directly reflecting the high concentration of these compounds.

#### Composition Profile of *Tillandsia usneoides* by Ultra-High-Performance Liquid Chromatography Coupled to a Diode Array Detector (UPLC-DAD)

**Figure 2:**
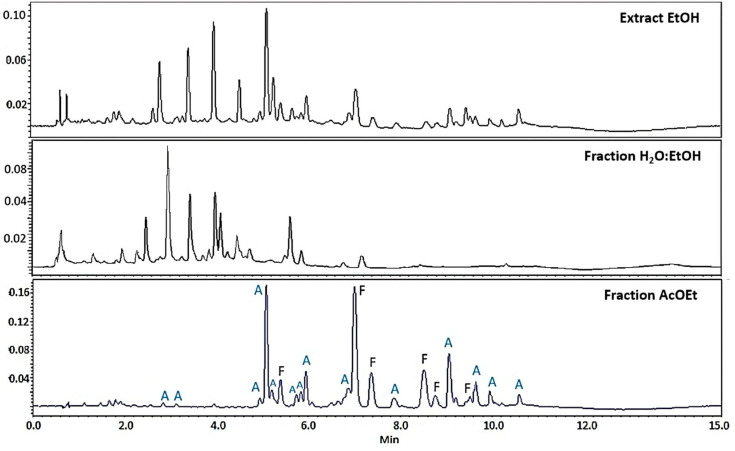
UPLC-DAD chromatographic profile at 254 nm of the EtOH extract and the H_2_O:EtOH and EtOAc fractions. 1.7 µm C18 column of 100 × 2.1 mm. Injection volume 3 µL. Flow rate 0.3 mL min^−1^. Mobile phase: acetonitrile and 0.1% formic acid gradient. A: phenolic acids. F: Flavonoids.

**Figure 3:**
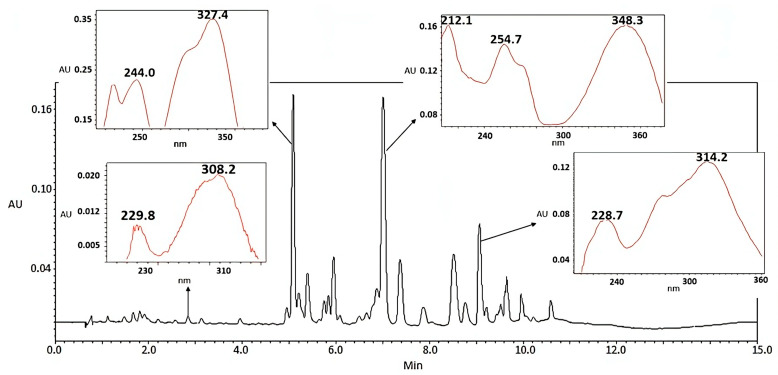
Maximum UV absorption spectra in the UPLC-DAD chromatogram at 254 nm of the EtOAc fraction. 1.7 µm C18 column of 100 × 2.1 mm. Injection volume 3 µL. Flow rate 0.3 mL min^−1^. Mobile phase: acetonitrile and 0.1% formic acid gradient.

To further characterize the *T. usneiodes* EtOH extracts and H_2_O:EtOH and EtOAc fractions, chromatographic profiles were obtained by UPLC-DAD at 254 nm (Figure 2). Both the EtOH extract and the H_2_O:EtOH fraction showed a similar profile, with a relatively higher abundance of flavonoids compared to phenolic acids, suggesting a variation in polarity and distribution of compounds between the two. In contrast, the EtOAc fraction exhibited a higher concentration of phenolic derivatives, including 14 phenolic acids and only 6 flavonoids (Figure 3).

### 2.2. Tillandsia Usneoides Extract, H_2_O:EtOH and EtOAc Fractions Did Not Affect the Cell Viability of Neurons In Vitro

**Figure 4:**
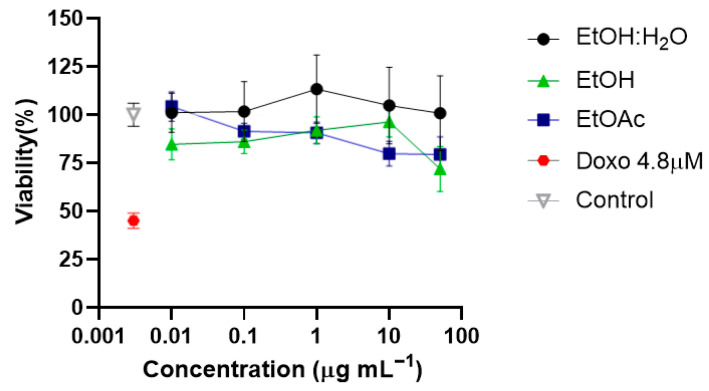
Cell viability of *Cortical Neurons* in vitro *treated with* the EtOH extract and the H_2_O:EtOH and EtOAc fractions from *T. usneoides*. Cortical neurons were seeded in 96-well plates at a density of ~2 × 10^4^ cells/well. Treatments were performed on day 6 of in vitro culture (DIV6). Control Vehicle: culture medium. Negative control: Doxorubicin at 4.8 μM (IC50). Concentrations: 0.01, 0.1, 1, 10, and 50 µg mL^−1^ (presented in Log 10), 95% confidence interval (CI), *n* = 12.

To evaluate the structural neuroplasticity-inducing potential of *T. usneoides*, we first assessed the dose–response effects of the H_2_O:EtOH and EtOAc fractions on cell viability using primary cortical neuron cultures derived from E18 rat embryos (Figure 4). The H_2_O:EtOH fraction did not affect cell viability at any tested concentration. Conversely, both the EtOH extract and the EtOAc fraction showed a dose–response relationship, with cell viability decreasing by approximately 20% at concentrations above 1 μg mL^−1^. Based on these findings, a concentration range of 0.01–1 μg mL^−1^ was selected for subsequent assays, as no significant effects on cell viability were observed within this range.

#### 2.2.1. Tillandsia Usneoides Increases the Dendritic Complexity of Cortical Neurons

**Figure 5:**
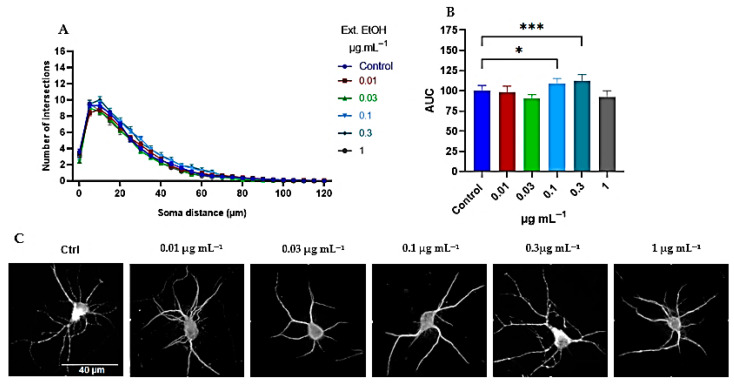
*T. usneoides* EtOH Extract Induces dendritogenesis in neurons. Neurons were seeded at a density of ~2 × 10^4^ cells/well, in 24-well plates with 8 mm coverslips previously treated with poly-D-lysine. On day DIV6, neurons were treated with the EtOH extract as well as the EtOAc and H_2_O:EtOH fractions at concentrations of 0.01, 0.03, 0.1, 0.3, and 1 μg mL^−1^, for 24 h. Immunocytochemical analysis was performed by labeling the cytoskeleton detecting the microtubule-associated protein 2 (MAP-2). Fluorescence images of MAP-2 labeling were obtained, and dendritic complexity was quantified by Sholl analysis. (**A**) The number of intersections (branches) of dendrites and their distance from the soma. (**B**) Area under the curve (AUC) and (**C**) representative images of neurons from each treatment group are also shown. Data are presented as mean ± SD. Asterisks indicate significant differences between the treatments and the control (untreated neurons), *n* = 75, CI 95.00%, *** *p* < 0.0002 and * *p* < 0.0431 (Tukey’s test). Concentration 0.3 μg mL^−1^, mean AUC = 112.

To study the effect of the EtOH extract and H_2_O:EtOH and EtOAc fractions of *T. usneoides* on neuronal structural plasticity, dendritic complexity and various morphological parameters were evaluated 24 h after the treatment. The Sholl analysis showed that the EtOH extract and both fractions affected the dendritic branching, although with different magnitudes. The EtOH extract showed a dose-dependent response. At low doses, it did not affect the number of dendrites; however, at 0.1 and 0.3 μg mL^−1^, there was a modest 8–12% increase in the area under the curve (AUC), suggesting an increase in the number of dendrites (Figure 5A–C). This stimulatory effect on dendritic branching disappeared at the 1 μg mL^−1^ dose (Figure 5A–C).

**Figure 6:**
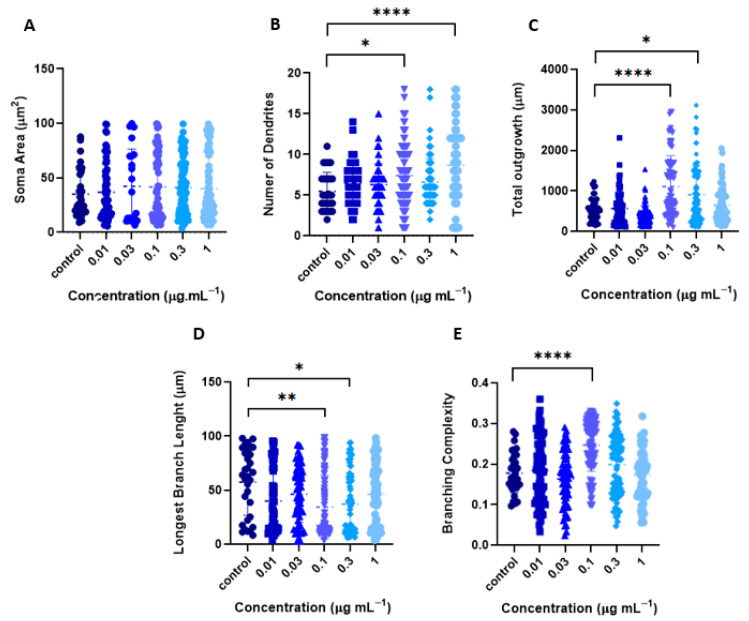
Morphometric parameters of cortical neurons induced by *T. usneoides* EtOH Extract. Neurons were seeded at a density of ~2 × 10^4^ cells/well, in 24-well plates with 8 mm coverslips previously treated with poly-D-lysine. On day DIV6, neurons were treated with the EtOH extract. Immunocytochemical analysis was performed by labeling the microtubule-associated protein 2 (MAP-2). Fluorescence images of MAP-2 labeling were obtained, and the morphometric parameters were determined using WISNeuromath software Version 3.4.8 and Matlab software Version R2023a with Synapse Detector (SynD). (**A**) Soma area (**B**) Number of dendrites. (**C**) Total outgrowth. (**D**) Length of longest dendrite. (**E**) Branching complexity (number of confirmed branches/length of confirmed dendrites) (**E**). **** *p* < 0.0001, ** *p* < 0.0016, * *p* < 0.0113 (Tukey’s test).

Morphological analysis of the soma and dendrites revealed that none of the EtOH concentrations affected soma size (Figure 6A). However, the number of primary dendrites (PD) significantly increased in neurons treated with 0.1 μg mL^−1^ (7.4 ± 0.4; *p* < 0.05) and 1 μg mL^−1^ (8.7 ± 0.6; *p* < 0.001), compared to the control group (5.5 ± 0.4) (one-way ANOVA followed by Tukey’s multiple comparisons test) (Figure 6B). Additionally, treatment with 0.1 (1105 ± 83.1 µm), 0.3 (904.4 ± 73.7 µm), and 1 μg mL^−1^ (655.4 ± 49.7 µm) significantly increased total dendritic outgrowth compared to the control (561.3 ± 44.8 µm) (Figure 6C). Despite this increase, a 28,9% reduction in the length of the longest branch was observed across all concentrations (Figure 6D), along with an increase in dendritic complexity or branching complexity, which refers to the number of branches per unit length of the dendrites [36], mainly at the 0.1 μg mL^−1^ dose (Figure 6E).

These results suggest that the *T. usneoides* EtOH extract modulates neuronal dendritic architecture in a dose-dependent manner. While it does not alter soma size, it increases the number of primary dendrites and total dendritic outgrowth, particularly at 0.1 μg mL^−1^, indicating a stimulatory effect on dendritogenesis. However, this is accompanied by a reduction in the length of the longest dendrite, suggesting a shift toward more complex but shorter branching.

**Figure 7:**
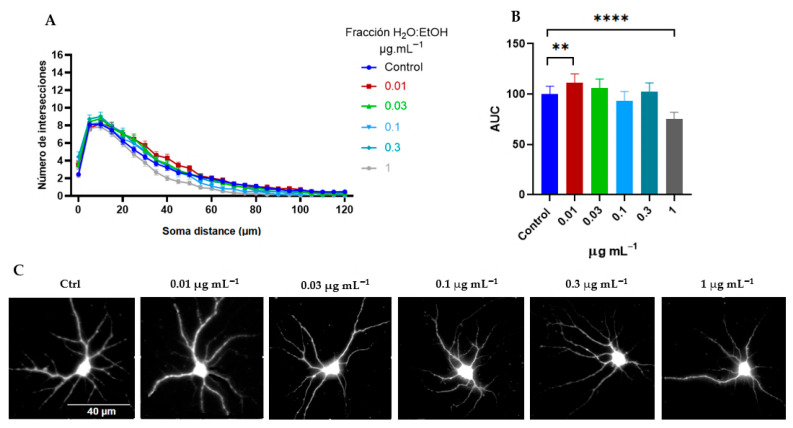
*T. usneoides* H_2_O:EtOH fraction induces dendritogenesis. Neurons were seeded at a density of ~2 × 10^4^ cells/well, in 24-well plates with 8 mm coverslips previously treated with poly-D-lysine. On day DIV6, neurons were treated with H_2_O:EtOH fraction at concentrations of 0.01, 0.03, 0.1, 0.3, and 1 μg mL^−1^, for 24 h. Immunocytochemical analysis was performed by labeling the microtubule-associated protein 2 (MAP-2). Fluorescence images of MAP-2 labeling were obtained, and dendritic complexity was quantified by Sholl analysis. (**A**) The number of intersections (branches) of dendrites and their distance from the soma. (**B**) Area under the curve (AUC) and (**C**) representative images of neurons from each treatment group are also shown. The asterisks represent significant differences between the treatments and the control (untreated neurons), *n* = 75, CI 95.00%, **** *p* < 0.0001, ** *p* < 0.0011 (Tukey’s test). Concentration 0.3 μg mL^−1^, mean AUC = 111.

**Figure 8:**
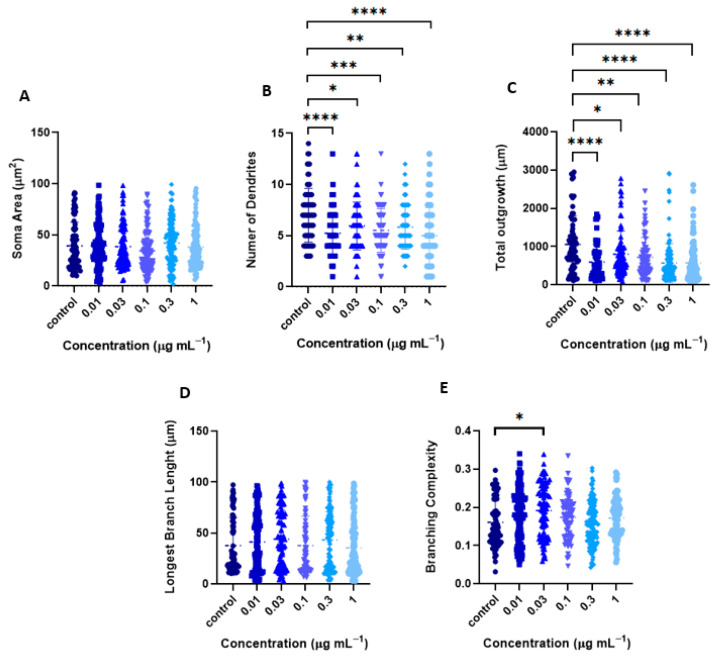
Changes in Morphometric parameters of cortical neurons induced by H_2_O:EtOH fraction of T. *usneoides*. Neurons were seeded at a density of ~2 × 10^4^ cells/well, in 24-well plates with 8 mm coverslips previously treated with poly-D-lysine. On day DIV6, neurons were treated with the H_2_O:EtOH fraction at concentrations of 0.01, 0.03, 0.1, 0.3, and 1 μg mL^−1^ for 24 h. Immunocytochemical analysis was performed by labeling the microtubule-associated protein 2 (MAP-2). Fluorescence images of MAP-2 labeling were obtained, and the morphometric parameters were determined using WISNeuromath software and Matlab software with Synapse Detector (SynD). (**A**) Soma area (**B**) Number of dendrites. (**C**) Total outgrowth. (**D**) Length of the longest dendrite. (**E**) Branching complexity (number of confirmed branches/length of confirmed dendrites). **** *p* < 0.0001, *** *p* < 0.0022, ** *p* < 0.0045, * *p* < 0.0361 (Tukey’s test).

In contrast, Sholl analysis of the H_2_O:EtOH fraction showed a dendritogenesis effect at a concentration of 0.01 μg mL^−1^ and a trend toward no effect or inhibition at higher concentrations (Figure 7A–C). Like the EtOH extract, the H_2_O:EtOH fraction did not affect the soma area (Figure 8A), however there is an inhibitory effect on the generation of primary dendrites. For instance, when compared with the control (7 ± 0.3), the 1 μg mL^−1^ showed a 29,5% decrease in the number of PD (5 ± 0.2,) (*p* < 0.001, one-way ANOVA followed by Tukey’s multiple comparisons test (Figure 8B). This inhibitory effect was also observed in all concentrations evaluated, although at different magnitudes (Figure 8B). Similarly, the H_2_O:EtOH fraction did not stimulate the dendritic growth and complexity (Figure 8C–E). Together, these results suggest that the H_2_O:EtOH fraction does not promote dendritic growth and branching and may exert an overall inhibitory effect on dendritic development.

**Figure 9:**
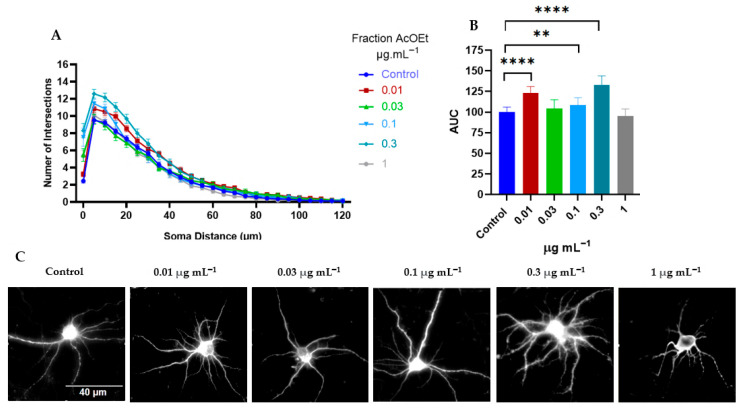
Differences in morphometric parameters of cortical neurons induced by *T. usneoides* EtOAc fraction. Neurons were seeded at a density of ~2 × 10^4^ cells/well, in 24-well plates with 8 mm coverslips previously treated with poly-D-lysine. On day DIV6, neurons were treated with EtOAc fraction at concentrations of 0.01, 0.03, 0.1, 0.3, and 1 μg mL^−1^, for 24 h. Immunocytochemical analysis was performed by labeling the microtubule-associated protein 2 (MAP-2). Fluorescence images of MAP-2 labeling were obtained, and dendritic complexity was quantified by Sholl analysis. (**A**) The number of intersections (branches) of dendrites and their distance from the soma. (**B**) Area under the curve (AUC) and (**C**) representative images of neurons from each treatment group are also shown. The asterisks represent significant differences between the different treatments and the control (untreated neurons), *n* = 75, CI 95.00%, **** *p* < 0.0001, ** *p* < 0.0015 (Tukey’s test), concentration 0.3 μg mL^−1^ AUC mean = 133.

**Figure 10:**
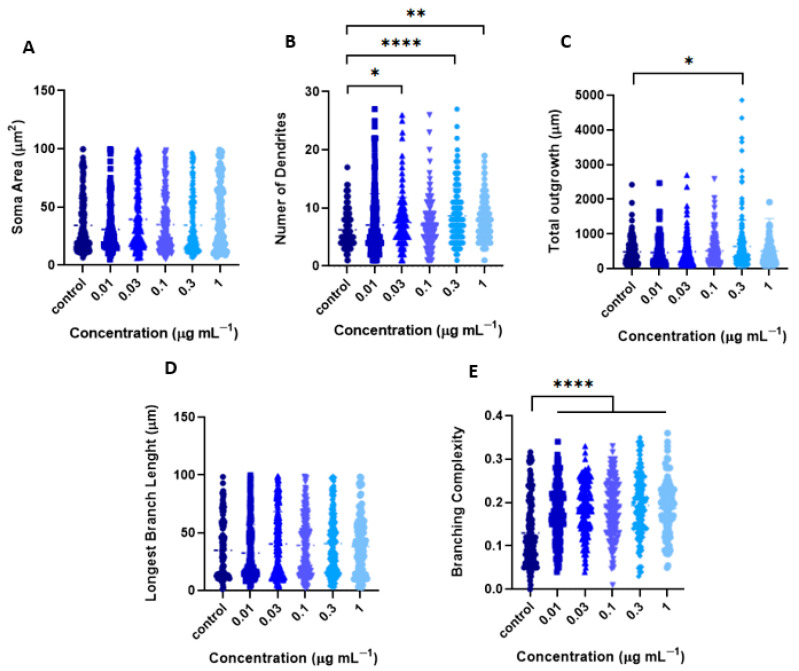
Morphometric parameters of cortical neurons induced by the EtOAc fraction from *T. usneoides*. Neurons were seeded at a density of ~2 × 10^4^ cells/well, in 24-well plates with 8 mm coverslips previously treated with poly-D-lysine. On day DIV6, neurons were treated with the EtOAc fraction at concentrations of 0.01, 0.03, 0.1, 0.3, and 1 μg mL^−1^ for 24 h. Immunocytochemical analysis was performed by labeling the microtubule-associated protein 2 (MAP-2). Fluorescence images of MAP-2 labeling were obtained, and the morphometric parameters were determined using WISNeuromath software and Matlab software with Synapse Detector (SynD). (**A**) Soma area (**B**) Number of dendrites. (**C**) Total outgrowth. (**D**) Length of the longest dendrite. (**E**) Branching complexity (number of confirmed branches/length of confirmed dendrites). * *p* < 0.0282, ** *p* < 0.0057, **** *p* < 0.0001 (Tukey’s test).

On the other hand, Sholl analysis of the EtOAc fraction showed that the 0.01 and 0.3 μg mL^−1^ concentrations significantly increased the number of dendritic branches compared to the control (Figure 9A–C). Additionally, while this fraction did not affect soma size (Figure 10A), it increased the number of primary dendrites at 0.03 μg mL^−1^ (7.51 ± 0.3), 0.3 μg/mL (8.7 ± 0.3), and 1 μg mL^−1^ (7.9 ± 0.3), compared to the control (6.3 ± 0.2) (*p*< 0.05, one-way ANOVA followed by Tukey’s multiple comparisons test (Figure 10B).

Although the EtOAc fraction stimulated increased dendritic arborization complexity at all concentrations tested (Figure 10E), only the 0.3 and 1 μg mL^−1^ doses significantly enhanced total dendritic outgrowth (Figure 10C), without affecting the length of the longest branch (Figure 10D). These findings suggest that the EtOAc fraction strongly promotes dendritic branching and complexity but does not enhance dendritic elongation.

**Figure 11:**
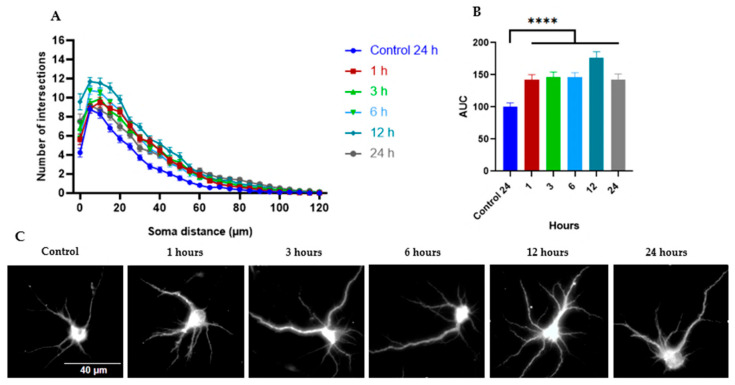
Time kinetics of dendritogenic effect induced by the EtOAc fraction from *T. usneoides*. Neurons were seeded at a density of ~2 × 10^4^ cells/well, in 24-well plates with 8 mm coverslips previously treated with poly-D-lysine. On day DIV6, neurons were treated with EtOAc fraction at a concentration of 0.3 μg mL^−1^ for 1, 3, 6, 12, and 24 h. Immunocytochemical analysis was performed by labeling the microtubule-associated protein 2 (MAP-2). Fluorescence images of MAP-2 labeling were obtained, and dendritic complexity was quantified by Sholl analysis. (**A**) The number of intersections (branches) of dendrites and their distance from the soma. (**B**) Area under the curve (AUC) and (**C**) representative images of neurons from each treatment group are also shown. **** *p* < 0.0001 (Tukey’s test).

The results taken together suggest that the EtOAc fraction induced a greater number of branches in cortical neurons (mean AUC of 133) (Figure 9A–C), compared to the whole extract (mean AUC of 112) and the H_2_O:EtOH fraction (mean AUC of 111) (Figure 5B and Figure 7B, respectively). For this reason, the time course kinetics of the EtOAc fraction at a concentration of 0.3 μg mL^−1^ was determined at different times. Figure 11 shows the results at 1, 3, 6, 12, and 24 h of treatment. It is observed that the dendritogenic effect begins early, starting at 1 hour, and remains constant over time. However, the time at which the greatest dendritic growth activity occurs is 12 h.

Although structural changes begin to appear within one hour of treatment (Figure 11), their maximum biological expression, which requires the synthesis of cytoskeletal proteins, transport proteins, and an increase in membranes and energy, occurs 12 h after treatment.

#### 2.2.2. *T. usneoides* Activates the ERK and PI3K Signaling Pathways Involved in the Induction of Dendritogenesis

Neuroplastic processes such as dendritogenesis have been linked to the activation of several kinases such as protein kinase C (PKC) [37], calcium-calmodulin kinase IV (CaMKIV) [38], and kinases associated with the PI3K and MAPK signaling pathways [39,40] that lead to the activation of a large group of genes, among which are those responsible for dendritic branching and properties [40]. To determine whether the EtOAc fraction can exert its neuroplasticity-inducing effect through the regulation of ERK and PI3K pathways. Was evaluated the phosphorylation of the ERK 1/2 and Akt 1/2 kinases using specific inhibitors of these pathways, PD98059 (MEK1 Inhibitor) and LY294002 (PI3K inhibitor).

Since the *Tillandsia usneoides* EtOAc fraction at 0.3 μg mL^−1^ induced the greatest increase in dendritic branching, neuronal cultures were treated for 1, 3, 6, 12, and 24 h to evaluate the potential involvement of the PI3K and MAPK signaling pathways in this stimulatory effect (Figure 11). These time points were assessed to identify the optimal window in which dendritic branching was enhanced by the EtOAc fraction without affecting cell viability during the application of specific inhibitors of these signaling pathways. Sholl analysis showed that the dendritogenic effect of the EtOAc fraction began as early as 1 h post-treatment and remained stable up to 6 h. This steady-state effect was followed by a significant increase in branching at 12 h, which was then followed by a reduction in branching after 24 h of treatment (Figure 11). Previous studies from our laboratory have shown that the PI3K and MAPK inhibitors LY294002 and PD98059 do not affect neuronal viability after a 6-hour treatment [41]. Therefore, the involvement of these signaling pathways was evaluated at this time point, when a significant increase in dendritic branching occurs without compromising neuronal viability.

**Figure 12:**
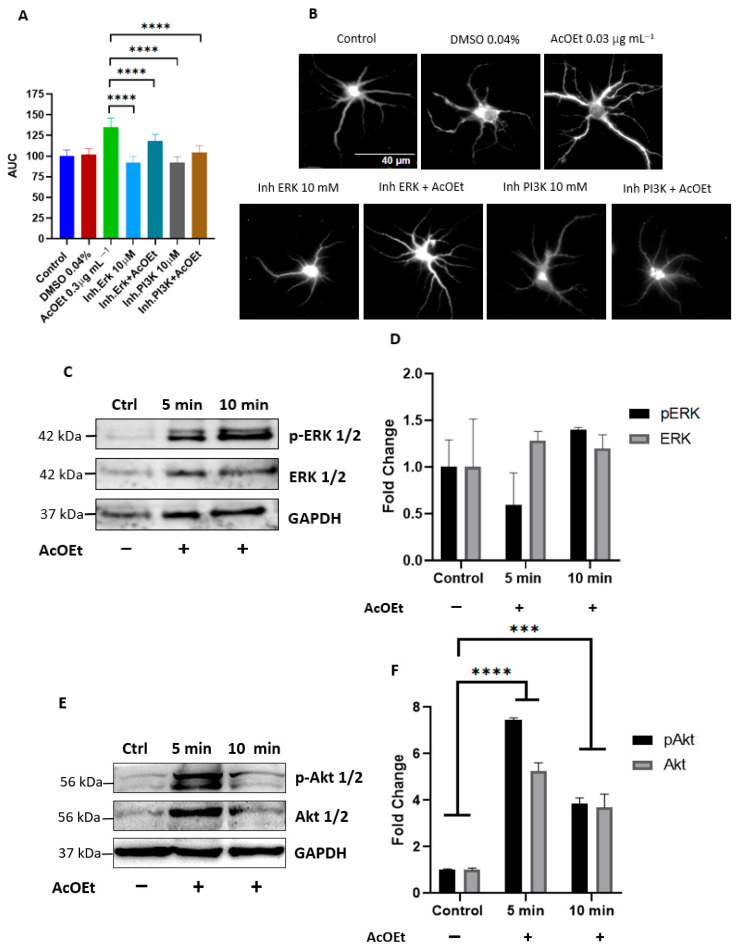
The EtOAc fraction induces neuronal dendritogenesis by activating PI3K/Akt and ERK 1/2 signaling pathways. Neurons were seeded at a density of ~2 × 10^4^ cells/well, in 24-well plates with 8 mm coverslips previously treated with poly-D-lysine. On day DIV6, neurons were treated with EtOAc fraction at a concentration of 0.3 μg mL^−1^ and the inhibitors. Inh.ERK: ERK inhibitor PD98059 (MEK1 Inhibitor), Inh.PI3K: PI3K pathway inhibitor LY294002 (PI3K inhibitor), 0.04% DMSO: vehicle control for the inhibitors, Control: untreated neurons, EtOAc + Inh: co-treatments of the EtOAc fraction 0.3 μg mL^−1^ with the inhibitors. Immunocytochemical analysis was performed by labeling the microtubule-associated protein 2 (MAP-2). Fluorescence images of MAP-2 labeling were obtained, and dendritic complexity was quantified by Sholl analysis. (**A**) Area under the curve (AUC), (**B**) Representative images of neurons from each treatment group are also shown, (**C**) Western blotting detection of activated proteins, ERK 1/2 kinase activation. (**D**) Relative differences in the expression of phosphorylated protein p-ERK 1/2, (**E**) Detection of activated proteins by Western blot, activation of Akt 1/2 kinase, (**F**) Relative differences in the expression of phosphorylated protein p-Akt 1/2. Phosphorylated protein expressions were normalized to control (untreated neurons) and loading control (GAPDH). MW: protein molecular weight markers. *n* = 85, CI 95.00%, *** *p* < 0.0002, **** *p* < 0.0001 (Tukey’s test).

Sholl analysis showed that the inhibitors LY294002 and PD98059 alone did not significantly affect dendritic branching compared to the control (Figure 12A,B). However, the dendritogenic effect of the *T. usneoides* EtOAc fraction was diminished when signaling pathway was inhibited. Inhibition of the PI3K pathway led to a 22.5% decrease in the area under the curve (AUC) compared to neurons treated with the EtOAc fraction alone (Figure 12A,B). Additionally, inhibition of the MAPK pathway also reduced dendritic branching, although to a lesser extent (12.3%). These results suggest that the dendritogenic effect of the EtOAc fraction involves the regulation of both signaling pathways.

To further confirm the involvement of the PI3K and MAPK pathways in the stimulatory effect of the EtOAc fraction, activation of these pathways was assessed by Western blot analysis. This was achieved by evaluating the phosphorylation levels of ERK1/2 and Akt1/2 proteins. Neurons treated with the EtOAc fraction for 5 and 10 min showed no changes in total ERK or pERK levels (Figure 12C,D). In contrast, treatment with the EtOAc fraction resulted in a sustained increase in both total Akt and pAkt levels compared to untreated neurons (Figure 12E,F). These results strongly suggest that the *T. usneoides* EtOAc fraction promotes dendritic branching primarily through activation of the PI3K signaling pathway.

## 3. Discussion

The complexity of the dendritic tree is essential for neuronal connectivity, synaptic integration, and plasticity, and its disruption is a hallmark of many neurodegenerative and neurodevelopmental conditions. The results of this study provide strong evidence that *Tillandsia usneoides*, particularly its EtOAc fraction, contains compounds capable of promoting structural neuroplasticity in rat primary cortical neurons.

Our chemical analysis revealed that *T. usneoides* contains a variety of secondary metabolites, including flavonoids, phenolic acids, sterols, and triterpenoids. These results are consistent with prior phytochemical characterizations of the *Tillandsia* genus, which report flavonoids (45%), triterpenes and steroids (51%), and phenolic acids (around 4%) as the main constituents [42]. Specifically, Cabrera et al. [30], Cabrera & Seldes [31] and Djerassi and McCrindle [43] identified several cycloartane-type triterpenes in *T. usneoides*, such as dimethyl 3,4-seco-cycloartane-4(29),24E-diene-3,26-diate, 27-nor-cycloartane-3,25-dione, and 3β-acetoxycycloartane-23-en-25-ol. These structures are characterized by the presence of a methylene bridge at positions 9 and 10. Moreover, the flavonoids previously reported, the predominant subgroups in terms of proportion are flavones (34%), flavonols (33%), anthocyanins (26%), flavanonols (4%), and flavonones (3%) [42].

HPTLC and UPLC-DAD analyses in this study confirmed the presence of these and other metabolites across solvent fractions with the EtOAc and EtOH fractions showing intense bands corresponding to flavonoids and phenolic acids. Flavonoids present two absorption maxima due to their two aromatic rings with conjugated double bonds. Ring A absorbs between 300 and 500 nm, and ring B comes from cinnamic acid and absorbs between 240 and 280 nm [44,45]. Except for the compound with a retention time (RT) of 8.5 min, whose absorption maximum is near 280 nm, typical of some chalcones that absorb at approximately 230, 280, and 320 nm [46,47]. On the other hand, phenolic acids generally show a UV absorption maximum near 280 nm, with a broad absorption range from 200 and 400 nm due to chemical diversity [48,49,50]. Thus, 70% of the compounds detected by UPLC-DAD were identified as phenolic acids, and 30% corresponded to flavonoids, with phenolic acids being proportionally more abundant in the EtOAc fraction, where the major compounds were those with retention times of 5.18 min and 7.0 min, a phenolic acid and a flavonoid, respectively.

These chemical features are highly relevant when considering the observed biological effects. Flavonoids and phenolic acids are known to possess neuroprotective properties, often attributed to their antioxidant, anti-inflammatory, anti-apoptotic activities and protection against glutamate-induced toxicity. Currently, research is being conducted to identify molecules that regulate neuroplastic processes that facilitate repair during injuries, as well as in pathological conditions [8,51,52]. For instance, cancer patients undergoing chemotherapy have been reported to experience memory loss, lack of concentration, and language difficulties, all symptoms related to cognitive impairment associated with the use of chemotherapy. Neurotoxicity caused by chemotherapeutics, such as doxorubicin, affects the structural and functional plasticity [52,53]. Several studies conducted on rat hippocampal neurons have suggested that some flavonoid-rich extracts exhibit neurotrophic activity as neurotrophin-like molecules and regulate various processes such as dendritic tree remodeling and spine number [13,16,54,55,56].

Our functional data show that the EtOAc fraction of *T. usneoides* significantly increases dendritic complexity at concentrations as low as 0.3 µg mL^−1^, without affecting neuronal viability. These results as related to the reported for other plant extracts ranging from 1 to 30 µg mL^−1^ [16,57]. The highest neuroplastic activity of *T. usneoides* coincided with the fraction that has the highest number of flavonoids, some of which have been reported to present different degrees of uncommon substitution, such as methoxylations and hydroxylations mainly at carbon 6 [58,59], suggesting that the characteristic substitution patterns (at carbons 6 and 8) for the species and the *Tillandsia* genus are due to phylogenetic advances as they result in an extra biosynthetic step [42,59].

These effects include increases in primary dendrite number, total dendritic outgrowth, and branch complexity, with no significant change in soma size. Inhibition experiments using LY294002 and PD98059 indicate that the effect is primarily mediated through the PI3K/Akt pathway, with a secondary contribution from the ERK/MAPK pathway. These results parallel those from other flavonoid studies where activation of these pathways is necessary for dendritic and synaptic remodeling [8,16,60,61,62]. It has been documented that some flavonoids such as flavan-3-ol (–)-epicatechin [63], 7,8-dihydroxyflavone [64], epigallocatechin gallate [65] and cyanidin [62], can induce the phosphorylation of ERK 1/2 and Akt 1/2. It has been suggested that the activation of these pathways occurs at specific concentrations of these types of compounds [66]. This effect could occur through the interaction of the Tropomyosin receptor Kinases receptor (TrkB) that activates the ERK pathway. PD98059 inhibits the MEK kinase upstream of the signaling cascade, which would explain the concentration-dependent activation [66,67,68,69].

Interestingly, while the EtOAc fraction showed potent effects, the H_2_O:EtOH fraction exhibited no stimulatory, despite also containing flavonoids. This divergence may reflect differences in the specific composition or relative abundance of active vs. inactive or antagonistic compounds in each fraction. As shown in previous studies, flavonoid bioactivity often depends on structural features such as the number and position of hydroxyl and methoxyl groups [59], as well as the presence of synergistic co-metabolites [57].

Temporal analysis of the EtOAc fraction’s effect revealed a rapid onset of action, beginning within 1 hour and peaking at 12 h, suggesting early activation of transcriptional and translational programs related to structural plasticity. This time course is consistent with the activation of CREB-mediated gene expression, which controls proteins essential for dendritic growth and maintenance [8,70,71,72]. The relatively sustained dendritic remodeling observed also supports previous reports that flavonoids can maintain morphological changes over time by inducing signaling-to-nucleus cascades requiring new protein synthesis [9,10,73].

It has also been proposed that certain flavonoids may interact directly with neurotrophin receptors such as TrkB, either enhancing the effects of endogenous neurotrophins or acting as agonists themselves [57,74,75]. This hypothesis aligns with our data showing that the EtOAc fraction’s effects are not purely concentration-dependent but instead follow a non-linear dose–response pattern. This suggests potential receptor saturation or differential activation at specific concentrations, a phenomenon also observed in prior studies of flavonoid-induced neuroplasticity [13,66].

The results of this study are consistent with the activating effects of structural neuroplasticity reported for flavonoid-type compounds [8,13,16]. On the other hand, it was found that the EtOAc fraction does not have an activity directly proportional to concentration; it is more active at a specific dose, which may be due to synergistic effects of the compounds in the fraction [57]. Therefore, the importance of knowing the chemical characterization of *T. usneoides* and the evaluation of the functional neuroplastic effects, as well as the identification of the cellular mechanisms associated with the activity of flavonoids, is highlighted.

## 4. Materials and Methods

### 4.1. Obtaining the Extract and Active Fraction

**Figure 13:**
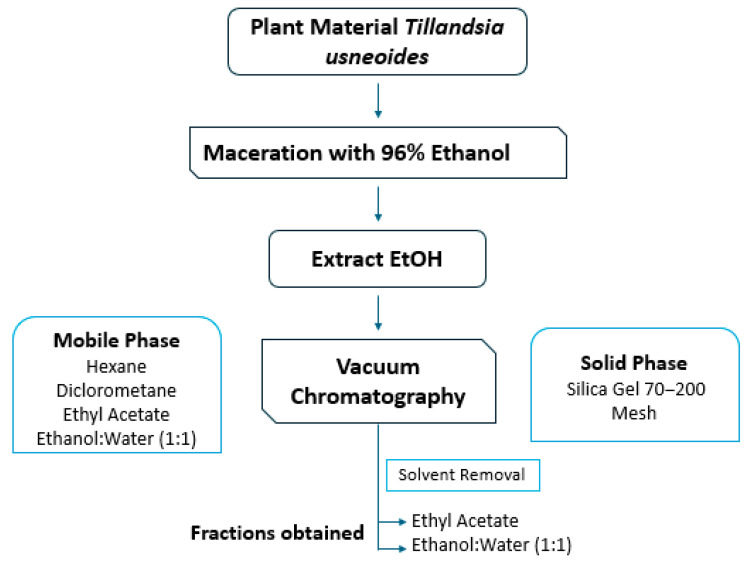
Flow diagram of fractionation of ethanol extract by preparative vacuum chromatography.

The vegetal material (leaves and stems of *Tillandsia usneoides*) was collected under the framework permit of the Pontificia Universidad Javeriana and the Contract for Access to Genetic Resources and Derived Products No. 212 (RGE 0287-6) and is indexed in the herbarium collection of the Pontificia Universidad Javeriana under registration number No. 30547. The plant material was collected in the city of Villa de Leyva, department of Boyacá, Colombia. The material was cleaned, dried in an oven with air circulation at 32 °C, and extracted by maceration with 96% ethanol, proportion 1:30 (*m*/*v*) (Figure 13). The extract obtained was fractionated by vacuum chromatography, using normal phase silica gel 70–200 Mesh as the stationary phase, and solvents of increasing polarity; hexane, dichloromethane (DCM), ethyl acetate (EtOAc) and ethanol/water (H_2_O:EtOH/1:1).

### 4.2. Qualitative Chemical Characterization

#### 4.2.1. High Performance Thin Layer Chromatography (HPTLC)

Chromatographic profiles of the extract and fractions were obtained by HPTLC using the automated HPTLC (Camag^®^ equipment, CAMAG, Muttenz, Switzerland). With silica gel 60 F254 HPTLC plates (10 × 20 cm; 200 μm) as stationary phase and as mobile phase the solvent system chloroform: acetone: formic acid (70:18:8 *v*/*v*) and as developing agent the Natural Reagent (2-aminoethyl diphenylborinate) + UV 365 nm, to identify phenolic derivatives. Additionally, the chromatographic profile was obtained to identify nonpolar compounds using as mobile phase: Toluene: Chloroform: Methanol (40:40:10) and developing with Vanillin in sulfuric acid/100 °C.

#### 4.2.2. Ultra-High-Performance Liquid Chromatography Coupled to a Diode Array Detector (UPLC-DAD)

Chromatographic analysis by UPLC-DAD was carried out on a Waters^®^ Acquity H Class instrument (Waters Corporation, Milford, MA, USA), using a 100 × 2.1 mm Phenome-nex^®^ Kinetex (Torrance, CA, USA)1.7 μm C18 silica column as stationary phase and a gradient of acetonitrile (A) and 0.1% formic acid (B) as mobile phase: 0–5 min (15–40% A), 5–7 min (40–40% A), 7–9 min (40–85% A), 9–10 min (85–85% A), 10–13 min (85–15% A) and 13–15 min (15–15% A), temperature 25 °C and flow 0.30 mL min^−1^. Chromatographic profiles of the EtOH extract and the H_2_O:EtOH and EtOAc fractions were obtained at 254 nm and 360 nm, along with ultraviolet (UV) spectra of each compound in a range of 200–600 nm.

### 4.3. Animals and Primary Neuron Culture

The neuron culture was performed from 18-day-old embryos (E18) of Wistar rats, after approval by the Institutional Committee for the Care and Use of Laboratory Animals of the Pontificia Universidad Javeriana (CICUA) (FUA-0057-18). The cortices of the brain’s embryos were dissected. The resulting tissues were incubated with trypsin (Trypsin-EDTA 1X) at 37 °C for 20 min and then resuspended in 10 mL of DMEM medium (Gibco™, Thermo Fisher Scientific, Waltham, MA, USA) supplemented with 10% fetal bovine serum at room temperature. The tissues were centrifuged at 1800 rpm for 5 min, discarding the supernatant and resuspending the pellet with Neurobasal medium (Gibco™). Neurons were maintained in Neurobasal medium supplemented with B27 1X factor (Gibco™) and GlutaMAX 1% (Gibco™) for 6 days in vitro (DIV6) before each experiment. The chemotherapy drug doxorubicin was used as a positive control for cell death, at a concentration of 4.8μM (IC50).

### 4.4. Cell Viability

To determine possible cytotoxic effects on cells, neuronal viability assays were performed using the MTT colorimetric technique, testing concentrations of 0.01–50 μg mL^−1^ of the EtOH extract and the EtOAc and H_2_O:EtOH fractions. Cortical neurons were seeded in 96-well plates at a density of ~2 × 10^4^ cells/well. Treatments were performed on day 6 of in vitro culture (DIV6). Each treatment was performed with 4 technical replicates (*n* = 12) and 3 biological replicates.

### 4.5. Evaluation of Dendritogenesis-Inducing Potential

Neurons were seeded at a density of ~2 × 10^4^ cells/well, in 24-well plates with 8 mm coverslips previously treated with poly-D-lysine. Neurons were treated on day DIV6 with the EtOH extract and the EtOAc and H_2_O:EtOH fractions at concentrations of 0.01, 0.03, 0.1, 0.3, and 1 μg mL^−1^, for 24 h. Each treatment included 2 technical replicates and 3 biological replicates, with a total of 75 neurons analyzed per treatment (*n* = 75 images). Subsequently, immunocytochemical analysis was performed by labeling the cytoskeleton by detecting the MAP-2 protein (associated with microtubules) [76], using the anti-MAP-2 primary antibody (Thermo polyclonal antibody 1:5000) at 4 °C overnight. Subsequently, the cells were washed with 1X PBS and incubated with the anti-mouse secondary antibody Alexa Fluor 568 (Thermo 1: 1000) for 1 hour at room temperature. For time-course kinetics analysis, neurons were seeded at a density of ~2 × 10^4^ cells/well in 24-well plates. On DIV6, neurons were treated with the EtOAc fraction at a concentration of 0.3 μg mL^−1^ for 1, 3, 6, 12, or 24 h. Each treatment had 2 technical replicates and 2 biological replicates. A total of 60 neurons were analyzed per treatment (*n* = 60 images). The acquired neurons were randomly taken from all visual fields, discarding those that presented defects in the immunolabeling. Immunocytochemical analysis was then performed following the previously described methodology.

Finally, the structural neuroplasticity inducing effect was observed, analyzing the morphological changes by fluorescence microscopy: number of branches, number of dendrites, length of primary dendrites, length of the longest dendrite, branching complexity, and soma area, qualitatively and quantitatively (morphometric analysis: Sholl) [20,41,77].

#### Image Acquisition and Processing

Fluorescence images of cell labeling were obtained with an AxioScope A1 epifluorescence microscope from Carl Zeiss and Zen 2 Lite Zeiss software. Images were transformed to high-resolution 8-bit TIFF format using Fiji software (ImageJ, Version 1.54). Morphometric parameters such as the number of dendrites, dendrite length, number of branches, soma area, and complexity index were determined using WISNeuromath software Version 3.4.8 and MATLAB software Version R2023a with Synapse Detector (SynD).

### 4.6. Determination of ERK and PI3K Pathway Activation by Western Blot Analysis

Cortical neurons were seeded at a density of ~6 *×* 10^6^ cells/well in 6-well plates. At DIV6, they were treated with 0.3 μg mL^−^^1^ of EtOAc for 5 and 10 min, except for the control (untreated neurons). Neurons were subsequently washed with cold 1X PBS twice for 5 min and lysed in cold RIPA buffer (RIPA Lysis Buffer System^®^ Santa Cruz Biotechnology Inc., Santa Cruz, CA, USA) for 15 min. Lysates were collected and seeded on 12% polyacrylamide gels for separation by SDS-PAGE. The separated proteins were then transferred to a PVDF membrane by wet transfer. Membranes with transferred proteins were blocked with 1% BSA in 0.1% TBS-T (0.1% Tween-20 in 1x TBS) for 90 min at room temperature with shaking. After transfer, the membranes were washed 3 times for 5 min with 0.1% TBS-T and then incubated with the primary antibodies pERK 1/2 (Thr202-Tyr204, Invitrogen), pAkt 1/2 (Rabbit Thr450, NOVUS; Rabbit Thr308, Cell Signaling), ERK 1/2 mouse, R&D Systems), Akt pan (mouse, R&D Systems) at 4 °C overnight. The primary antibody was removed from the membranes, and these were washed with 0.1% TBST, 3 times for 5 min. The membranes were incubated with horseradish peroxidase (HRP)-conjugated secondary antibodies to finally identify the target proteins using the developer reagent. Semi-quantitative densitometric analysis was performed using ImageJ software.

### 4.7. Statistical Analysis

For all biological assays, in addition to descriptive statistics for each experiment, where means are expressed with the standard error of the mean (SEM) or standard deviation (SD), two-way ANOVA parametric tests were performed to assess the effects and significant differences between treatments, using a significance level of *p* < 0.05. Post hoc comparisons were conducted using Tukey’s test (*p* < 0.0001). Statistical analyses were performed using GraphPad Prism 8^®^ software.

## 5. Conclusions

Phenolic acids and flavonoids were identified from *Tillandsia usneoides.* Additionally, terpenoid and sterol compounds were found to be abundant in the medium-polarity fractions. It was determined that the ethanolic extract and the EtOAc fraction did not exhibit cytotoxic effects on rat cortical neurons. Both the extract and the EtOAc fraction demonstrated structural neuroplasticity–induction activity, increasing the number of branches and the size of dendrites at concentrations between 0.01 and 0.3 μg mL^−1^, mainly after 12 h of treatment. These results suggest that the compounds present in the active fraction may regulate processes related to neuronal neuroplasticity, specifically dendritogenesis through the regulation of the ERK 1/2 and Akt pathways. These results are promising for the identification of potential alternatives that protect against cognitive decline in its early stages. However, further investigation of these dendritogenic effects in in vivo models of cognitive impairment or in models of neurodegenerative diseases.

## Data Availability

The raw data supporting the conclusions of this article will be made available by the authors on request.

## References

[1] Martin R.E. (2009). Neuroplasticity and Swallowing. Dysphagia.

[2] Garcés M.V., Suárez N.C. (2014). Neuroplasticidad: Aspectos bioquímicos y neurofisiológicos. CES Med..

[3] Johnston M.V. (2009). Plasticity in the developing brain: Implications for rehabilitation. Dev. Disabil. Res. Rev..

[4] Hummel F.C., Cohen L.G. (2005). Drivers of brain plasticity. Curr. Opin. Neurol..

[5] Popescu B.O., Batzu L., Ruiz P.J.G., Tulbă D., Moro E., Santens P. (2024). Neuroplasticity in Parkinson’s disease. J. Neural Transm..

[6] Gulyaeva N.V. (2017). Molecular mechanisms of neuroplasticity: An expanding universe. Biochemistry.

[7] Mishra A., Patni P., Hegde S., Aleya L., Tewari D. (2020). Neuroplasticity and environment: A pharmacotherapeutic approach towards preclinical and clinical understanding. Curr. Opin. Environ. Sci. Health.

[8] Cichon N., Saluk-Bijak J., Gorniak L., Przyslo L., Bijak M. (2020). Flavonoids as a natural enhancer of neuroplasticity-an overview of the mechanism of neurorestorative action. Antioxidants.

[9] Platholi J., Lee F.S. (2018). Neurotrophic Factors. Handbook of Developmental Neurotoxicology.

[10] Citri A., Malenka R.C. (2008). Synaptic plasticity: Multiple forms, functions, and mechanisms. Neuropsychopharmacology.

[11] Jaworski J., Spangler S., Seeburg D.P., Hoogenraad C.C., Sheng M. (2005). Control of dendritic arborization by the phosphoinositide-3′-kinase- Akt-mammalian target of rapamycin pathway. J. Neurosci..

[12] Reiner A., Levitz J. (2018). Glutamatergic Signaling in the Central Nervous System: Ionotropic and Metabotropic Receptors in Concert. Neuron.

[13] Bhuiyan M.M.H., Haque M.N., Mohibbullah M., Kim Y.K., Moon I.S. (2017). *Radix Puerariae* modulates glutamatergic synaptic architecture and potentiates functional synaptic plasticity in primary hippocampal neurons. J. Ethnopharmacol..

[14] Ongnok B., Chattipakorn N., Chattipakorn S.C. (2020). Doxorubicin and cisplatin induced cognitive impairment: The possible mechanisms and interventions. Exp. Neurol..

[15] Ramalingayya G.V., Cheruku S.P., Nayak P., Kishore A., Shenoy R., Rao C.M., Krishnadas N. (2017). Rutin protects against neuronal damage in vitro and ameliorates doxorubicin-induced memory deficits in vivo in Wistar rats. Drug Des. Devel. Ther..

[16] Bhuiyan M.M.H., Mohibbullah M., Hannan M.A., Hong Y.K., Choi J.S., Choi I.S., Moon I.S. (2015). *Undaria pinnatifida* promotes spinogenesis and synaptogenesis and potentiates functional presynaptic plasticity in hippocampal neurons. Am. J. Chin. Med..

[17] Cho J.H., Jung J.Y., Lee B.J., Lee K., Park J.W., Bu Y. (2017). Epimedii Herba: A Promising Herbal Medicine for Neuroplasticity. Phytother. Res..

[18] Ip P.S.P., Tsim K.W.K., Chan K., Bauer R. (2012). Application of complementary and alternative medicine on neurodegenerative disorders: Current status and future prospects. J. Evid. Based Complement. Altern. Med..

[19] Ramalingayya G.V., Nampoothiri M., Nayak P.G., Kishore A., Shenoy R.R., Rao C.M., Nandakumar K. (2016). Naringin and rutin alleviates episodic memory deficits in two differentially challenged object recognition tasks. Pharmacogn. Mag..

[20] Mitra S., Munni Y.A., Dash R., Sultana A., Moon I.S. (2022). Unveiling the effect of *Withania somnifera* on neuronal cytoarchitecture and synaptogenesis: A combined in vitro and network pharmacology approach. Phytother. Res..

[21] Wang Y., Wang L., Wu J., Cai J. (2006). The in vivo synaptic plasticity mechanism of EGb 761-induced enhancement of spatial learning and memory in aged rats. Br. J. Pharmacol..

[22] Williams R.J., Spencer J.P.E., Rice-Evans C. (2004). Flavonoids: Antioxidants or signalling molecules?. Free Radic. Biol. Med..

[23] Shaker F.H., El-Derany M.O., Wahdan S.A., El-Demerdash E., El-Mesallamy H.O. (2021). Berberine ameliorates doxorubicin-induced cognitive impairment (chemobrain) in rats. Life Sci..

[24] Tannock I.F., Ahles T.A., Ganz P.A., van Dam F.S. (2004). Cognitive impairment associated with chemotherapy for cancer: Report of a workshop. J. Clin. Oncol..

[25] Papini A., Tani G., Di Falco P., Brighigna L. (2010). The ultrastructure of the development of *Tillandsia* (Bromeliaceae) trichome. Flora—Morphology, Distribution. Funct. Ecol. Plants..

[26] Estrella E., Flores M., Blancas G., Koch S., Alarcón F. (2019). The *Tillandsia* genus: History, uses, chemistry, and biological activity. J. Bol. Latinoam. Caribe Plantas Med. Aromát..

[27] Garth R.E. (1964). The Ecology of Spanish Moss (*Tillandsia Usneoides*): Its Growth and Distribution. Ecology.

[28] Vargas W. (2002). Guía Ilustrada De Las Plantas de Las Montañas Del Quindio Y Los Andes Centrales.

[29] Pineda A.G. (2006). Flora Útil: Etnobotánica de Nicaragua.

[30] Cabrera G.M., Gallo M., Seldes A.M. (1995). A 3,4-seco-cycloartane derivative from *Tillandsia usneoides*. Phytochemistry.

[31] Cabrera G.M., Seldes A.M. (1997). Short side-chain cycloartanes from *Tillandsia usneoides*. Phytochemistry.

[32] Rodriguez A. (2021). Evaluación Del Efecto Neuroprotector Y de Los Cambios en La Complejidad Dendrítica Inducidos Por Los Extractos de Tillandsia usneoides Y Lippia alba en Cultivo Primario de Neuronas Tratadas Con Agentes Quimioterapéuticos. Tesis de Maestría.

[33] Lewis D., Mabry T.J. (1977). 3,6,3′,5′-tetrametroxy-5,7,4′-trihydroxyflavone from *Tillandsia usneoides*. Phytochemistry.

[34] Wollenweber E., Mann K., Roitman J.N. (1992). A Myricetin Tetramethyl Ether from the Leaf and Stem Surfaces of *Tillandsia usneoides*. Z. Für Naturforsch..

[35] Wagner H., Bladt S. (1996). A Thin Layer Chromatography Atlas.

[36] Rishal I., Golani O., Rajman M., Costa B., Ben-Yaakov K., Schoenmann Z., Yaron A., Basri R., Fainzilber M., Galun M. (2012). WIS-neuromath enables versatile high throughput analyses of neuronal processes. Dev. Neurobiol..

[37] Alkon D.L., Sun M.K., Nelson T.J. (2007). signaling deficits: A mechanistic hypothesis for the origins of Alzheimer’s disease. Trends Pharmacol. Sci..

[38] Wei F., Qiu C.S., Liauw J., Robinson D.A., Ho N., Chatila T., Zhuo M. (2002). Calcium calmodulin-dependent protein kinase IV is required for fear memory. Nat. Neurosci..

[39] Rojas P.S., Aguayo F., Neira D., Tejos M., Aliaga E., Muñoz J.P., Parra C.S., Fiedler J.L. (2017). Dual effect of serotonin on the dendritic growth of cultured hippocampal neurons: Involvement of 5-HT_1A_ and 5-HT_7_ receptors. Mol. Cell Neurosci..

[40] Spencer J.P., Vauzour D., Rendeiro C. (2009). Flavonoids and cognition: The molecular mechanisms underlying their behavioural effects. Arch. Biochem. Biophys..

[41] Velásquez M.M., Lattig M.C., Chitiva L.C., Costa G.M., Sutachan J.J., Albarracin S.L. (2023). Dendritogenic Potential of the Ethanol Extract from *Lippia alba* Leaves in Rat Cortical Neurons. Molecules.

[42] Vasconcelos A.L., Vasconcelos A.L., Ximenez E.A., Randau K.P. (2013). *Tillandsia recurvata* L. (Bromeliaceae): Aspectos farmacognósticos. Rev. Ciênc Farm. Básica Apl..

[43] Djerassi C., McCrindle R. (1962). Terpenoids Part LI The isolation of some new cyclopropanecontaining triterpenes from Spanish moss (*Tillandsia usneoides* L). J. Chem. Soc..

[44] Nascimento L.B., Leal-Costa M.B., Menezes E., Rodrigues V., Muzitano M., Costa S., Schwartz E. (2015). Ultraviolet-B radiation effects on phenolic profile and flavonoid content of *Kalanchoe pinnata*. J. Photochem. Photobiol. B.

[45] Dewick P.M. (2009). Medicinal Natural Products: A Biosynthetic Approach.

[46] Minsat L., Peyrot C., Brunissen F., Renault J.H., Allais F. (2021). Synthesis of Biobased Phloretin Analogues: An Access to Antioxidant and Anti-Tyrosinase Compounds for Cosmetic Applications. Antioxidants.

[47] Abbo H.S., Hung L.C., Titinchi S.J.J. (2023). Substituent and solvent effects on UV-visible absorption spectra of chalcones derivatives: Experimental and computational studies. Spectrochim. Acta A Mol. Biomol. Spectrosc..

[48] Kowalski R., Kowalska G. (2005). PHENOLIC ACID CONTENTS IN FRUITS OF AUBERGINE (*Solanum melongena* L.). Pol. J. Food Nutr. Sci..

[49] Souza M., Comin J., Moresco R., Maraschin M., Kurtz C., Lovato E.P., Lourenzi R.C., Pilatti K.F., Loss A., Kuhnen S. (2021). Exploratory and discriminant analysis of plant phenolic profiles obtained by UV-vis scanning spectroscopy. J. Integr. Bioinform..

[50] Kaeswurm J.A.H., Scharinger A., Teipel J., Buchweitz M. (2021). Absorption Coefficients of Phenolic Structures in Different Solvents Routinely Used for Experiments. Molecules.

[51] Semwal P., Kapoor T., Anthwal P., Sati B.K., Thapliyal A. (2014). Herbal Extract as Potential Modulator and Drug for Synaptic Plasticity and Neurodegenerative Disorders. Int. J. Pharm. Sci. Rev..

[52] Rendeiro C., Rhodes J.S., Spencer J.P. (2015). The mechanisms of action of flavonoids in the brain: Direct versus indirect effects. Neurochem. Int..

[53] Flanagan E., Müller M., Hornberger M., Vauzour D. (2018). Impact of Flavonoids on Cellular and Molecular Mechanisms Underlying Age-Related Cognitive Decline and Neurodegeneration. Curr. Nutr. Rep..

[54] Urbanska M., Blazejczyk M., Jaworski J. (2008). Molecular basis of dendritic arborization. Acta Neurobiol. Exp..

[55] Hannan M.A., Kang J.Y., Hong Y.K., Lee H.S., Chowdhury M.T.H., Choi J.S., Choi I.S., Moon I.S. (2012). A brown alga *Sargassum fulvellum* facilitates neuronal maturation and synaptogenesis. In Vitro Cell Dev. Biol. Anim..

[56] Hannan M.A., Mohibbullah M., Hong Y.K., Nam J.H., Moon I.S. (2014). *Gelidium amansii* promotes dendritic spine morphology and synaptogenesis, and modulates NMDA receptor-mediated postsynaptic current. Vitr. Cell Dev. Biol. Anim..

[57] Xia C.X., Gao A.X., Dong T.T., Tsim K.W. (2023). Flavonoids from Seabuckthorn (*Hippophae rhamnoides* L.) mimic neurotrophic functions in inducing neurite outgrowth in cultured neurons: Signaling via PI3K/Akt and ERK pathways. Phytomedicine.

[58] Williams C.A. (1978). The systematic implications of the complexity of leaf flavonoids in the bromeliaceae. Phytochemistry.

[59] Manetti L.M., Delaporte R.H., Laverde A. (2009). Metabólitos Secundários da Família Bromeliaceae. Quim. Nova..

[60] Sharma P., Kumar A., Singh D. (2019). Dietary Flavonoids Interaction with CREB-BDNF Pathway: An Unconventional Approach for Comprehensive Management of Epilepsy. Curr. Neuropharmacol..

[61] Numakawa T., Richards M., Nakajima S., Adachi N., Furuta M., Odaka H., Kunugi H. (2014). The role of brain-derived neurotrophic factor in comorbid depression: Possible linkage with steroid hormones, cytokines, and nutrition. Front. Psychiatry.

[62] Shan X., Chen J., Dai S., Wang J., Huang Z., Lv Z., Qian W., Wu Q. (2020). Cyanidin-related Antidepressant-like Efficacy Requires PI3K/AKT/FoxG1/FGF-2 Pathway Modulated Enhancement of Neuronal Differentiation and Dendritic Maturation. Phytomedicin.

[63] Schroeter H., Bahia P., Spencer J.P.E., Sheppard O., Rattray M., Rice-Evans C., Williams R.J. (2007). (–)-epicatechin stimulates ERK-dependent cyclic AMP response element activity and upregulates GLUR2 in cortical neurons. J. Neurochem..

[64] Gao L., Tian M., Zhao H.Y., Xu Q.Q., Huang Y.M., Si Q.C., Tian Q., Wu Q.M., Hu X.M., Sun L.B. (2016). TrkB activation by 7, 8-dihydroxyflavone increases synapse AMPA subunits and ameliorates spatial memory deficits in a mouse model of Alzheimer’s disease. J. Neurochem..

[65] Ding M.L., Ma H., Man Y.G., Lv H.Y. (2017). Protective effects of a green tea polyphenol, epigallocatechin-3-gallate, against sevoflurane-induced neuronal apoptosis involve regulation of CREB/BDNF/TrkB and PI3K/Akt/mTOR signalling pathways in neonatal mice. Can. J. Physiol. Pharmacol..

[66] Spencer J.P. (2007). The interactions of flavonoids within neuronal signalling pathways. Genes Nutr..

[67] Alessi D.R., Cuenda A., Cohen P., Dudley D.T., Saltiel A.R. (1995). PD 098059 is a specific inhibitor of the activation of mitogen-activated protein kinase kinase in vitro and in vivo. J. Biol. Chem..

[68] Schroeter H., Boyd C., Spencer J.P., Williams R.J., Cadenas E., Rice-Evans C. (2002). MAPK signaling in neurodegeneration: Influences of flavonoids and of nitric oxide. Neurobiol. Aging.

[69] Schroeter H., Spencer J.P., Rice-Evans C., Williams R.J. (2001). Flavonoids protect neurons from oxidized low-density-lipoprotein-induced apoptosis involving c-Jun N-terminal kinase (JNK), c-Jun and caspase-3. Biochem. J..

[70] Ha S., Redmond L. (2008). ERK mediates activity dependent neuronal complexity via sustained activity and CREB-mediated signaling. Dev. Neurobiol..

[71] Mendoza M.C., Er E.E., Blenis J. (2011). The Ras-ERK and PI3K-mTOR pathways: Cross-talk and compensation. Trends Biochem. Sci..

[72] Brivio P., Sbrini G., Corsini G., Paladini M.S., Racagni G., Molteni R., Calabrese F. (2020). Chronic Restraint Stress Inhibits the Response to a Second Hit in Adult Male Rats: A Role for BDNF Signaling. Int. J. Mol. Sci..

[73] Thomas G.M., Huganir R.L. (2004). MAPK cascade signalling and synaptic plasticity. Nat. Rev. Neurosci..

[74] Spencer J.P., Schroeter H., Crossthwaithe A.J., Kuhnle G., Williams R.J., Rice-Evans C. (2001). Contrasting influences of glucuronidation and O -methylation of epicatechin on hydrogen peroxide-induced cell death in neurons and fibroblasts. Free Radic. Biol. Med..

[75] Spencer J.P., Abd-el-Mohsen M.M., Rice-Evans C. (2004). Cellular uptake and metabolism of flavonoids and their metabolites: Implications for their bioactivity. Arch. Biochem. Biophys..

[76] DeGiosio R.A., Grubisha M.J., MacDonald M.L., McKinney B.C., Camacho C.J., Sweet R.A. (2022). More than a marker: Potential pathogenic functions of MAP2. Front. Mol. Neurosci..

[77] Binley K.E., Ng W.S., Tribble J.R., Song B., Morgan J.E. (2014). Sholl analysis: A quantitative comparison of semi-automated methods. J. Neurosci. Methods.

